# Advanced Optical Materials: From Materials to Applications

**DOI:** 10.3390/ijms242115790

**Published:** 2023-10-31

**Authors:** Bong-Hyun Jun

**Affiliations:** Department of Bioscience and Biotechnology, Konkuk University, Seoul 143-701, Republic of Korea; bjun@konkuk.ac.kr

Optical materials interact significantly with electromagnetic radiation in the visible, ultraviolet, and infrared regions of the spectrum. They find use in a wide variety of applications, including bio-applications, OLEDs, and solar cells [1,2,3,4,5]. The field of advanced optical materials is rapidly evolving, with the continuous development of new materials offering numerous advantages over traditional ones, such as improved performance and greater flexibility. In this Special Issue, seven papers discussing developments related to advanced optical materials are presented.

In the realm of nanotechnology for bio-applications, which encompasses bioimaging, diagnostics, novel optical probes, and therapeutic agents, there is ongoing research on advanced optical materials [6,7,8,9]. These materials play a crucial role in enhancing the capabilities of various biomedical applications, enabling a higher sensitivity, resolution, and specificity. One of the key areas of interest is the development of advanced nanomaterials for bioimaging [10].

Quantum dots (QDs) are promising fluorescent probes for bioimaging due to their excellent optical properties [11]. To enhance the fluorescence signal of QDs, novel nanoparticles (NPs) consisting of a silica core, multiple embedded QDs, and a silica shell were developed [7,12,13,14]. However, Cd-based QDs, which have been widely studied, have potential toxicity problems. Ham et al. reported the fabrication of Cd-free QD nanoprobes for bioimaging [15] by densely embedding multiple indium phosphide/zinc sulfide (InP/ZnS) QDs onto silica templates and coating them with a silica shell. The resulting silica-coated InP/ZnS QD-embedded silica NPs (SiO_2_@InP QDs@SiO_2_ NPs) exhibited hydrophilic properties and high brightness, with a quantum yield (QY) of 6.61% and a full-width half-maximum (FWHM) of 44.62 nm. The authors also demonstrated that the SiO_2_@InP QDs@SiO_2_ NPs could be used for bioimaging in tumor syngeneic mice, with the fluorescence signal prominently detected in the tumors. These results suggest that SiO_2_@InP QDs@SiO_2_ NPs have the potential to replace Cd-based QDs as highly bright and biocompatible fluorescent nanoprobes.

Another significant aspect of research involves diagnostic applications using advanced optical materials. A review using optical NPs presented in this Special Issue provides insight into how to advance previous studies and suggests methods of applying optical nanoparticle-based sensors. The review by Kim et al. introduces the recent trends in lateral flow immunoassays (LFIAs) with optical NPs [16]. LFIA is a promising diagnostic tool because it is simple, rapid, and low-cost. However, its sensitivity and reproducibility have been limited. Recent advances in the engineering of optical NPs have laid the foundations for more sensitive and accurate LFIAs. The paper discusses LFIA systems with various types of optical NPs, including Au NPs, carbon NPs, QDs, UCNPs, and silica template-based NPs. These systems have shown effectiveness in detecting clinically relevant targets, such as cancer biomarkers. The paper also highlights the potential of LFIA for biomarker detection at an early disease stage, even when analyzing a small amount of sample.

Gálico et al. reported the development of a bifunctional temperature and oxygen dual probe based on anthracene and europium complex luminescence [17]. The probe is a polydimethylsiloxane membrane that contains two emitter groups, anthracene and [Eu(bzac)_3_], which are chemically attached to the membrane structure. The ratio of the anthracene and europium(III) emission components can be used to measure temperature and oxygen levels. The authors found that the probe is operational in the 203–323 K range, with an observed maximum relative sensitivity of 2.06% K^−1^ at 290 K. The probe can also measure oxygen levels at 25, 30, 35, and 40 °C. The authors’ findings suggest that the probe is a promising tool for temperature and oxygen sensing. It is also interesting to note that the probe exhibits anthracene → europium(III) energy transfer, even though there is no chemical bonding between the two species.

Furthermore, the advancement of optical materials is also driving progress in therapies. The paper by Baek et al. investigates the effect of electrical stimulation on the osteogenic differentiation of human adipose-derived mesenchymal stem cells (hASCs) on O_2_ plasma-treated ITO glass [18]. hASCs are a promising cell source for bone tissue engineering. Electrical stimulation has been shown to be effective in promoting bone healing, but the mechanisms by which it works are not fully understood. The authors found that electrical stimulation with an amplitude of 10 µA or 50 µA at a frequency of 10 Hz promoted osteogenic differentiation of hASCs on O_2_ plasma-treated ITO glass. This was evident by increased expression of osteogenic markers, such as Runx2 and ALP. The authors also found that electrical stimulation enhanced the adhesion and proliferation of hASCs on O_2_ plasma-treated ITO glass. The authors’ findings suggest that electrical stimulation is a promising strategy for promoting osteogenic differentiation of hASCs, which could be used to develop new treatments for bone defects.

This Special Issue also introduces research related to the applications of OLED (Organic Light-Emitting Diode) technology, in addition to the nanobio field. Popielarski et al. investigated the effect of annealing on the optical properties of nickel and copper phthalocyanine thin films [19]. Phthalocyanines are organic compounds with a wide range of potential applications in optoelectronic devices, such as solar cells, sensors, displays, and OLEDs. The authors found that annealing the films can change their optical properties, such as the refractive index, extinction coefficient, and absorption coefficient. The authors also found that annealing can change the morphology of the films, as seen in AFM images. Raman measurements showed that the band at about 1526 cm^−1^ (B1g symmetry) has a higher intensity for the α form than for the β form. The intensity of this band is related to changing the form of phthalocyanine from α to β. The authors’ findings suggest that annealing can be used to tune the optical properties of phthalocyanine thin films, which could be used to improve the performance of optoelectronic devices.

Lypenko et al. reported a study on Cu (II) protoporphyrin and chlorin as materials for near-IR OLEDs [20]. The authors fabricated OLEDs based on a composite containing a hole-transporting PVK and an electron-transporting PBD doped with 1–5 wt. % of either Cu (II) protoporphyrin Cu-PP-IX or chlorin Cu-C-e6. The devices exhibited UV and IR EL emission spectra, with the IR emission of the chlorin Cu-C-e6-containing OLEDs spanning up to 1100 nm. The authors found that protoporphyrin is more promising for OLED development than chlorin, as it exhibits enhanced emissions in the near-IR range compared to the UV range of the spectrum. The charge carrier mobility in the Cu-PP-IX and Cu-C-e6 solid layers was also measured for the first time. Both materials possess reasonable electron and hole mobilities on the order of 10^−5^ cm^2^ V^−1^ s^−1^. The authors’ findings suggest that Cu (II) protoporphyrin and chlorin are promising materials for near-IR OLEDs, which could be used to develop new types of OLEDs that emit in the near-IR range, with applications in various fields such as medical imaging and security.

The Special Issue also introduces potential optical materials. Pach-Zawada et al. reported the effects of doping tellurite glasses with erbium ions on the structure and physical properties of the glasses [21]. The results show that doping with erbium ions causes changes in the parameters describing the structure of the glasses, including the positron trapping rate and the fraction of trapped positrons. Raman spectroscopy revealed that the introduction of Er^3+^ ions into the structure of the tellurite glass breaks the Te-O-Te bridges and leads to the formation of new Te-O-Er bonds. The results of Faraday effect measurements indicate the possibility of changing the magneto-optical properties of tellurite glasses by the incorporation of various modifiers into the network and doping with rare earth metal ions. In conclusion, the paper finds that doping of tellurite glasses with erbium ions has significant effects on the micro- and nanostructural properties of the glasses. These effects could be exploited to tailor the properties of tellurite glasses for specific applications.

In this Special Issue highlight the significant progress that has been made in the field of advanced optical materials in recent years. These materials offer a wide range of potential applications, and the field is poised for continued growth in the years to come. Some of the key challenges that need to be addressed in the future include the following: the development of new methods for the synthesis and characterization of advanced optical materials; the optimization of the properties of advanced optical materials for specific applications; and the development of new processing techniques for the fabrication of advanced optical devices. The papers in this Special Issue have made significant contributions to the field of advanced optical materials. Their work provides a solid foundation for future research and development in this exciting field.
